# Genome-Wide Search for Genes Required for Bifidobacterial Growth under Iron-Limitation

**DOI:** 10.3389/fmicb.2017.00964

**Published:** 2017-05-31

**Authors:** Noreen Lanigan, Francesca Bottacini, Pat G. Casey, Mary O'Connell Motherway, Douwe van Sinderen

**Affiliations:** APC Microbiome Institute and School of Microbiology, University College CorkCork, Ireland

**Keywords:** ferrous, ferric, ferric reductase, siderophore, Fe-S cluster

## Abstract

Bacteria evolved over millennia in the presence of the vital micronutrient iron. Iron is involved in numerous processes within the cell and is essential for nearly all living organisms. The importance of iron to the survival of bacteria is obvious from the large variety of mechanisms by which iron may be acquired from the environment. Random mutagenesis and global gene expression profiling led to the identification of a number of genes, which are essential for *Bifidobacterium breve* UCC2003 survival under iron-restrictive conditions. These genes encode, among others, Fe-S cluster-associated proteins, a possible ferric iron reductase, a number of cell wall-associated proteins, and various DNA replication and repair proteins. In addition, our study identified several presumed iron uptake systems which were shown to be essential for *B. breve* UCC2003 growth under conditions of either ferric and/or ferrous iron chelation. Of these, two gene clusters encoding putative iron-uptake systems, *bfeUO* and *sifABCDE*, were further characterised, indicating that *sifABCDE* is involved in ferrous iron transport, while the *bfeUO*-encoded transport system imports both ferrous and ferric iron. Transcription studies showed that *bfeUO* and *sifABCDE* constitute two separate transcriptional units that are induced upon dipyridyl-mediated iron limitation. In the anaerobic gastrointestinal environment ferrous iron is presumed to be of most relevance, though a mutation in the *sifABCDE* cluster does not affect *B. breve* UCC2003's ability to colonise the gut of a murine model.

## Introduction

Iron is an essential nutrient for nearly all forms of life, having co-evolved with biological systems for billions of years; it is a key element in many redox reactions and is involved in various cellular processes such as DNA replication, nitrogen fixation, nucleotide biosynthesis and the production of various metabolites (Ilbert and Bonnefoy, 2013). The prevalence and importance of iron in biological systems is most likely due to the abundance of Fe^2+^ during the initial evolution of living organisms (Crichton and Pierre, 2001; Ilbert and Bonnefoy, 2013). The ionic forms of iron found in nature represent two interchangeable redox states: ferrous iron (Fe^2+^) and its more oxidized form ferric iron (Fe^3+^). These attributes make iron a versatile prosthetic component in many proteins as a biocatalyst or electron carrier (Andrews et al., 2003). Under aerobic neutral pH conditions iron is present in its oxidized, but essentially insoluble trivalent, ferric form, while under anaerobic, neutral pH conditions iron is found in its reduced, divalent ferrous state, the latter being more biologically accessible due to its greater solubility (Kortman et al., 2014).

Iron speciation, which relates to the changing concentration of varying forms of an ion as the pH of the solution changes, and iron availability are two factors which are important for the accessibility of iron within the gastrointestinal environment. Iron availability can vary greatly along the length of the gastrointestinal tract due to its tendency to complex with other molecules and because of its ability to exist in various oxidation states depending on its surrounding environment (Ilbert and Bonnefoy, 2013). The low pH in the stomach favors the solubility of both ferric and ferrous iron, whereas the subsequent rise in pH upon entry into the small intestine results in a decrease in the solubility of ferric iron. Iron may then complex with food components or host/microbiota-derived compounds such as citrate, ascorbate, mucin, certain amino acids, or lactate (Andrews et al., 2003). Certain insoluble forms of ferric iron, for example when complexed with phosphate, carbonate or oxides, are not readily available to the microbiota and may require reduction or removal from these complexes by siderophore activity (Kortman et al., 2014). Soluble forms of ferric iron may be reduced to ferrous iron upon entry into the cytoplasm, while ferrous citrate or ferrous ascorbate may be taken up directly by (certain components of) the microbiota. Finally, lactate and short chain fatty acid (SCFA) production by the microbiota may also cause a modest, possibly localised drop in the pH in the colon resulting in an increase in iron solubility (den Besten et al., 2013).

The human gut microbiota typically includes clostridia, eubacteria, and species of the genera *Bacteroides* and *Bifidobacterium* (Picard et al., 2005). Of particular interest, the genus *Bifidobacterium* belongs to the *Bifidobacteriaceae* family, which in turn belongs to the *Actinobacteria* phylum. Henri Tissier was the first to isolate a *Bifidobacterium* species in 1899 from the faeces of a healthy, breast-fed infant, and due to his pioneering work and subsequent research of many others, members of this genus are today considered to represent health-promoting or probiotic bacteria (Sanders, 2008).

Competitive sequestration/withholding of iron has long been known in both Gram-positive and -negative bacteria as a defense mechanism, conferring a competitive advantage to certain commensals, such as bifidobacteria which thrive in low iron environments (Collins, 2008; Dostal et al., 2015). Bacteria have developed a wide range of mechanisms for iron sequestration, including a variety of systems for the uptake of ferrous iron, ferric iron, heme, hemephores (which scavenge heme from various hemoproteins), and siderophores (selective iron chelators which have a high affinity for ferric iron). Iron sequestration via siderophore-mediated and/or direct iron uptake has been reported in the *Bifidobacterium* genus, with *Bifidobacterium breve* exhibiting the highest siderophore activity and *Bifidobacterium kashiwanohense* exhibiting the highest iron uptake (Hood and Skaar, 2012; Vazquez-Gutierrez et al., 2015). Furthermore, bifidobacterial carbohydrate fermentation typically acidifies its surrounding environment, favouring the solubility of ferric and ferrous iron in a localised fashion (Vernazza et al., 2006). Interestingly, certain species of *Bifidobacterium* have also been found to bind ferric iron to their cell wall and membranes, thereby limiting its availability to other bacteria (Kot and Bezkorovainy, 1999). Bifidobacteria, like many other bacteria, are known to import iron (ions) across the cytoplasmic membrane by means of ABC-type transporters (Brown and Holden, 2002). In the case of *B. breve*, this leads to a cytoplasmic iron concentration (100/200 μM) which is about 14–16 times higher than its surrounding environment (Kot et al., 1993; Brown and Holden, 2002). Global gene expression analysis of *B. breve* UCC2003 under iron-limiting conditions has also revealed a number of iron-responsive genes, including *bfeUOB* (Cronin et al., 2012). The *bfeUOB* gene cluster is homologous to the *Escherichia coli efeUOB* genes, which are known to encode an iron transport system. The promoter of *bfeUOB* was found to be inducible under iron-limiting conditions and was utilised to create an inducible promoter system in bifidobacteria. A subsequent study found that insertional mutants created in the *bfeUO* genes and adjacent genes (with locus tags Bbr_0223-Bbr_0227) caused a decreased ability of *B. breve* to confer protection against *Salmonella* infection in the *C. elegans* model and to survive iron limitation (Christiaen et al., 2014).

In the current study random mutagenis was combined with global transcriptional profiling and led to the identification of a number of genes which are vital for *B. breve* UCC2003 survival under ferrous and/or feric iron limitiation. The identified genes are predicted to represent a variety of iron-related functions, such as a ferric iron reductase, Fe-S cluster associated proteins, a number of cell wall associated proteins, several DNA replication/repair proteins and various transport systems.

## Materials and methods

### Bacterial strains and culture conditions

Bacterial strains and plasmids used in this study are listed in Table 1. *B. breve* UCC2003 was routinely grown at 37°C in either de Man Rogosa and Sharpe medium (MRS medium; Difco, BD, Le Pont de Claix, France), modified de Man Rogosa and Sharpe (mMRS) medium made from first principles (Man et al., 1960), or reinforced clostridial medium (RCM; Oxoid Ltd., Basingstoke, Hampshire, United Kingdom) supplemented with 0.05% cysteine-HCl. Iron-limiting experiments were carried out in filtered RCM (fRCM) prepared in de-mineralised water. Maximal tolerable concentration (MTC) of dipyridyl, ciclopirox olamine, and phenanthroline were 275, 100, and 80 μM respectively, as determined by Christiaen et al. (2014). Bifidobacterial cultures were incubated anaerobically in a modular, atmosphere-controlled system (Davidson and Hardy, Belfast, Ireland). Where appropriate growth medium was supplemented with tetracycline (Tet; 10 μg ml^−1^), chloramphenicol (Cm; 5 μg ml^−1^ for *L. lactis* and *E. coli*, 2.5 μg ml^−1^ for *B. breve*), ampicillin (Amp; 100 μg ml^−1^), erythromycin (Em; 100 μg ml^−1^) or kanamycin (Kan; 50 μg ml^−1^) for plasmid selection and maintenance.

**Table 1:** Bacterial strains and plasmids used in this study.

**Strains and plasmids**	**Relevant features**	**References or source**
**STRAINS**		
***B. breve***		
UCC2003	Isolate from nursling stool	Law et al., 1995
UCC2003-*bfeU*	pORI19-*tetW*-*bfeU* insertion mutant of UCC2003	Christiaen et al., 2014
UCC2003-*sifA*	pORI19-*tetW*-*sifA* insertion mutant of UCC2003	Christiaen et al., 2014
UCC2003-pK1	UCC2003 harboring pPKCM	O'Connell Motherway et al., 2011
***E. coli***		
XL1 BLUE	supE44 hsdR17 recA1 gyrA96 thi relA1 lac F = [proAB laclq lacZ M15 Tn10(Tet^*r*^)]	Stratagene
EC101	Cloning host; *repA*^+^ *km^*r*^*	Law et al., 1995
*L. lactis* NZ9000	MG1363, pepN::nisRK, nisin-inducible overexpression host	de Ruyter et al., 1996
**PLASMIDS**		
pORI19	Em^*r*^, repA^−^, ori^+^, cloning vector	Law et al., 1995
pORI19-*bfeU*	pOR19 harboring internal fragment of *bfeU* (*Bbr_0221*)	Christiaen et al., 2014
pORI19-*bfeU*-tet	pORI19 harboring internal fragment of *Bbr_0221* + Tet^*r*^	Christiaen et al., 2014
pORI19-*sifA*	pOR19 harboring internal fragment of *bfeB* (*Bbr_0223*)	Christiaen et al., 2014
pORI19-*sifA*-tet	pORI19 harboring internal fragment of *bfeB* + Tet^*r*^	Christiaen et al., 2014
pBC1.2	pBC1-pSC101-Cmr	Alvarez-Martin et al., 2007
pBC1.2-bfeU-IR	pBC1.2 harboring *bfeU* promoter	This study
pBC1.2-*sifA*-IR	pBC1.2 harboring *sifA* promoter	This study
pNZ272	Cm^*r*^, pSH71 derivative containing promoterless glucuronidase gene for promoter screening	Platteeuw et al., 1994
pNZ272-*bfeU*-IR	pNZ272 derivative carrying the *bfeU* promoter	This study
pNZ272-*sifA*-IR	pNZ272 derivative carrying the *sifA* promoter	This study

### Phenotypic screening and monitoring of dipyridyl-sensitive growth

Phenotypic screening and determination of transposon insertion sites was carried out as described previously (Ruiz et al., 2013) with the following adjustments: *B. breve* UCC2003 transposon mutants were sub-cultured twice in RCM supplemented with tetracycline and spotted onto Q-Trays (Molecular Devices, Berkshire, United Kingdom) containing RCA or RCA supplemented with the iron chelator dipyridyl at a concentration of either 250 or 275 μM. These two different concentrations of dipyridyl were chosen as they did not impair growth of the WT strain and to reduce the number of false positives identified in subsequent confirmatory analysis. Transposon mutants which grew on RCA, but failed to grow or showed poor growth on RCA supplemented with (either concentration of) dipyridyl were then selected for further analysis. The phenotype of such dipyridyl-sensitive mutants was confirmed by monitoring growth in fRCM and fRCM containing dipyridyl (at a final concentration of 250 or 275 μM). Such mutants were incubated anaerobically at 37°C, with OD_600_ readings taken at 24 h following inoculation. Mutants which were found to be sensitive to growth on dipyridyl were further tested for growth on fRCM in the presence of ciclopirox olamine (100 μM) and phenanthroline (80 μM).

### Nucleotide sequence analysis

Sequence data was obtained from the Artemis-mediated (Rutherford et al., 2000) genome annotations of the *B. breve* UCC2003 genome sequence (O'Connell Motherway et al., 2011). Data base searches were carried out using non-redundant sequences accessible at the National Centre for Biotechnology Information internet site (https://www.ncbi.nlm.nih.gov/) utilising the basic alignment search tool (Blast). Sequence analysis was performed by using the Seqbuilder and Seqman programs of the DNASTAR software package (DNASTAR, Madison, WI). Protein functions were assigned with the use of the basic protein alignment search tool BlastP and homology detection and structure prediction by HMM-HMM comparison; HHpred (Altschul et al., 1990, 1997). Rho-independent terminators were identified utilising ARNold (Gautheret and Lambert, 2001). Bacterial promoters were predicted with BPROM (http://softberry.com).

### DNA manipulations

DNA manipulations were carried out as previously reported (Russell, 2001). Restriction enzymes and T4 DNA ligase were obtained from Roche Diagnostics, and were used according to the manufacturer's instructions. PCRs were performed using Extensor Long Range PCR Enzyme master mix (Thermo Scientific). Synthetic oligonucleotides were synthesized by Eurofins (Ebersberg, Germany) and are listed in Table 2. PCR products were purified by the use of a High-Pure PCR product purification kit (Roche). Plasmid DNA was introduced into *E. coli* and *B. breve* by electroporation and large-scale preparation of chromosomal DNA from *B. breve* was performed as described previously (O'Riordan and Fitzgerald, 1999). Plasmid DNA was obtained from *B. breve* and *E. coli* using the Roche High Pure plasmid isolation kit (Roche Diagnostics, Basel, Switzerland). An initial lysis step was performed using 30 mg ml^−1^ of lysozyme for 30 min at 37°C as part of the plasmid purification protocol for *B. breve*.

**Table 2:** Oligonucleotide primers used in this study.

**Purpose**	**Primer**	**Sequence**
Cloning *bfeU* promotor in pNZ272	*bfeU*-GUS-F	ATAGCTGGATCCGAGATCTGTCCGTTGGCGCTG
	*bfeU*-GUS-R	ATAGCTGAATTCGAAGGAATCGGCAACGTG
Cloning *sifA* promotor in pNZ272	*sifA*-GUS-F	ATAGCTGGATCCTCGACAACTGGGACTACACC
	*sifA*-GUS-R	ATAGCTGAATTCCATCACGCTCAGGCACATCAC
Cloning *bfeU* promoter in pBC1.2	*bfeU*-PE-F	ATAGCTCTGCAGGAGATCTGTCCGTTGGCGCTG
	*bfeU*-PE-R	CCTGACTCTAGAGATGGCCTTGGACACGTC
Cloning *sifA* promoter in pBC1.2	*sifA*-PE-F	ATAGCTCTGCAGTCGACAACTGGGACTACACC
	*sifA*-PE-R	CCTGACTCTAGAAGCACCGGATAGTTGACGAA
RT-PCR Bbr_0220-Bbr_0228	Bbr_0220-RT-1	AAGTGCGTGCCATGATGATC
	*bfeU*-RT-2	CAACGTGCGTATCGTGTTCT
	*bfeU*-RT-3	CTCCATGGCTGTTTCGATGG
	*bfeO*-RT-4	CCATCGAAACAGCCATGGAG
	*bfeO*-RT-5	TCGACAACTGGGACTACACC
	sifA-RT-6	TGCCACGAATTGTTCAAGCA
	*sifA*-RT-7	CGATTCCGTTCCCGTACAAG
	*sifB*-RT-8	CATACGGTAAGCGCGATGAG
	*sifB*-RT-9	GATTCTGAACCTCAAGCCCG
	*sifC*-RT-10	CATCCTGAAGAACATGCCGG
	*sifC*-RT-11	CGCTCCATCGGATTCAACTG
	*sifD*-RT-12	CGGTCAGATTGAGGTCGTCT
	*sifD*-RT-13	GCGAGAAGTGGAATGACGAG
	*sifE*-RT-14	GCTCTTCCTGCGATTTCTGG
	*sifE*-RT-15	GGTCTTTGGTGGCGTATGAGG
	Bbr_0228-RT-16	CCGTTCACCAAGATTTCCAAGG
Sequencing primers, Tn5 random mutant library	pMOD-fw-seq	GCCAACGACTACGCACTAGCC
	pMOD-rev-seq	GAGCCAATATGCGAGAACACC
Inverse PCR primers, Tn5 random mutant library	i-PCR-fw	GCATACCGTACTGATCTG
	i-PCR-rev	CAATCATACCGGCTTCC
IRD700 primers, primer extension products	*bfeU*ifF	GAAGGAATCGGCAACGTG
	*bfeU*ifR	TCAATGCGAACAGGAACACGAC
	*bfeO*ifF	GACGAACCGCAAGCAGCC
	*bfeO*ifR	CTTCTTGGCGGTGTCGGAG
	*sifAi*fF	CTCAGGCACATCACCAGTAA
	*sifA*ifR	GCCGATGAGACGCCAATG

### Transcriptome analysis

*B. breve* UCC2003's transcriptome response to iron limitation was tested by subjecting exponentially growing cells to a high concentration (700 μM) of dipyridyl for 30 min. This concentration was chosen in order to rapidly and substantially reduce iron availability, thus allowing the monitoring of an immediate transcriptional response of *B. breve* UCC2003 to iron limitation. Cells were prepared as follows; an overnight culture of *B. breve* UCC2003 in RCM was inoculated at 1% into filtered RCM broth and incubated until an OD_600_ of 0.5 was reached. Cells were then exposed to 700 μM dipyridyl for 30 min. *B. breve* UCC2003 controls were treated in the same manner except for the addition of dipyridyl. Following dipyridyl exposure as outlined above (or in the absence of dipyridyl for controls), cells were harvested by centrifugation at 10,000 rpm for 2 min at room temperature and immediately frozen at −80°C prior to RNA isolation. DNA microarrays containing oligonucleotide primers representing each of the annotated genes on the genome of *B. breve* UCC2003 were designed by and obtained from Agilent Technologies (Palo Alto, CA, USA). Cell disruption, RNA isolation, RNA quality control, and cDNA synthesis and labeling were performed as described previously (Zomer et al., 2009). The labeled cDNA was hybridized using the Agilent Gene Expression hybridization kit (part number 5188–5242) as described in the Agilent Two-ColorMicroarrayBased Gene Expression Analysis v4.0 manual (G4140-90050). Following hybridization, the microarrays were washed in accordance with Agilent's standard procedures and scanned using an Agilent DNA microarray scanner (model G2565A). The generated scans were converted to data files with Agilent's Feature Extraction software (v9.5). The DNA microarray data were processed as previously described (García de la Nava et al., 2003; van Hijum et al., 2003, 2005). Differential expression tests were performed with the Cyber-T implementation of a variant of the *t*-test (Long et al., 2001). A gene was considered to exhibit a significantly different expression level relative to the control when *p* < 0.001 and an expression ratio of > 1.7 or < 0.25. The microarray data obtained in this study have been deposited in NCBI's Gene Expression Omnibus database and is accessible through GEO series accession number GSE92758.

### Real time-PCR

Primers were chosen to amplify upstream regions of all genes from Bbr_0220-Bbr_0227 (Table 1). *B. breve* UCC2003 was prepared for RNA extraction as follows: cells were incubated until an OD_600_ of 0.5 was achieved and were then exposed to 700 μM dipyridyl for 30 min, cells were harvested by centrifugation at 10,000 rpm for 2 min at room temperature and immediately frozen at −80°C. Cell disruption, RNA isolation and cDNA synthesis were performed as previously described (Gueimonde et al., 2009). Two microgram of RNA was treated with 2 units of DNase, RNase free (Roche) for 1 h at 37°C. cDNA was synthesized using Superscript III reverse transcriptase (Invitrogen). Absence of chromosomal DNA contamination was checked by real-time PCR. RT-PCR experiments were carried out using the cDNA-containing sample as a template and employing primers as above and Extensor Hi-Fidelity PCR Master Mix (Thermo Scientific).

### Beta-glucoronidase assay

The potential promoter-containing regions of *bfeU* and *sifA* were amplified by PCR using primer combinations *bfeU*-GUS-F, *bfeU*-GUS-R, and *sifA*-GUS-F, *sifA*-GUS-R (Table 2), which contain EcoRI and BamH1 restriction sites at their 5′ ends. Amplicons were digested with EcoRI and BamH1, and cloned upstream of the promoterless *gusA* gene present in the similarly restricted pNZ272 reporter vector (Platteeuw et al., 1994). Ligation mixtures were introduced by electroporation in *Lactococcus lactis* NZ9000 competent cells. The resulting plasmids, pNZ272-bfeU-IR and pNZ272-sifA-IR, once verified by restriction and sequence analysis, were then introduced into *B. breve* UCC2003 by electroporation (Table 1). GusA activity assays in *B. breve* UCC2003 were carried out in triplicate by independent assay as previously described (Alvarez-Martin et al., 2012), with the following modifications: cells were grown in fRCM to an OD_600_ of ~0.3–0.4, at which transcription was induced by the addition of 700 μM dipyridyl for 20 min. Two hundred microliters of cell culture were used in the assay. GusA activity was expressed in Miller Units and calculated using the following equation: 1000^*^((O.D._420_ − (1.75^*^OD_550_))/(t × v × O.D._600_)), where t is reaction time in minutes, v is cell volume in [ml] and OD_420_,OD_550_, and OD_600_ are absorbance values at 420, 550, and 600 nm.

### Primer extensions

The upstream (intergenic) regions of *bfeU* and *sifA* were PCR amplified using *B. breve* UCC2003 genomic DNA as the template and oligonucleotide primer combinations listed in Table 3. These PCR products were then individually ligated into pBC1.2, using PstI and XbaI restriction sites and transformed into *E. coli* XL1 BLUE cells by electroporation. *E. coli* XL1 BLUE transformants containing the recombinant pBC1.2 constructs were selected for on LB agar with appropriate antibiotics. The integrity of the constructs was then confirmed by restriction and sequence analysis, and plasmid preparations of resulting recombinant plasmids, designated pBC1.2-*bfeU*-IR and pBC1.2-*sifA*-IR (names correspond to the gene downstream of the promoter in the UCC2003 genome, see Table 1), were introduced by electroporation into *B. breve* UCC2003 with selection on RCA supplemented with the appropriate antibiotic.

**Table 3:** Transposon mutants identified under iron limiting conditions and their growth profile.

**Gene**	**Predicted function**	**No chelator**	**250 μM dipyridyl^a^**	**275 μM dipyridyl^a^**	**100 μM ciclopirox olamine^b^**	**80 μM phenanthroline^b^**	**Upregulation transcriptome analysis^c^**
**TRANSPORT ASSOCIATED**							
UCC2003	*B. breve* UCC2003 WT strain	+++	+++	+++	+++	+++	N/A
Bbr_0221	*bfeU*, high affinity Fe^2+^ periplasmic transporter	+++	–	–	–	–	Yes
Bbr_0222	*bfeO*, high affinity Fe^2+^ periplasmic transporter	+++	–	–	–	–	Yes
Bbr_0223	*sifA*, possible siderophore binding protein	+++	–	–	+++	–	Yes
Bbr_0224^d^	*sifB*, permease protein, ferric iron/siderophore uptake	+++	–	–	+++	–	Yes
Bbr_0225^d^	*sifC*, permease protein, ferric iron/siderophore uptake	+++	–	–	+++	–	Yes
Bbr_0226^e^	*sifD*, heme ATP binding domain, heme ABC transporter	+++	–	–	+++	–	No
Bbr_0227	*sifE*, possible ferric iron reductase, FMN binding domain	+++	–	–	+++	+	No
Bbr_0427	*mntH*, Mn^2+^/Fe^2+^ transporter, NRAMP family	+++	+	–	+++	++	No
Bbr_1656	Sugar ABC transporter, permease protein	+++	++	++	++	++	No
Bbr_1657	Binding-protein-dependent transport system	+++	++	++	++	++	No
Bbr_1669	Hypothetical membrane spanning protein	++	+	–	++	–	No
Bbr_1816	*oppC*, permease protein iron siderophore uptake	+++	+++	++	+++	++	Yes
**Fe-S CLUSTER ASSEMBLY ASSOCIATED**							
Bbr_0669	*apbC*, Iron-sulfur cluster binding protein	++	+	–	–	–	No
Bbr_1825	*apbE*, Iron-sulfur cluster formation/repair	+++	+	+	+++	–	No
**CELL ENVELOPE ASSOCIATED**							
Bbr_0246	*murE*, UDP-N-acetylmuramoylalanyl-D-glutamate–2,6-diaminopimelate ligase	+++	++	++	+	–	No
Bbr_1592	Putative lipid kinase	+++	++	++	++	–	No
Bbr_1916	Hypothetical secreted protein, in operon with peptidoglycan lipid II flipase	+++	+	++	+	–	No
**DNA REPAIR/REPLICATION**							
Bbr_0511	Alanine aminopeptidase	+++	++	++	++	++	No
Bbr_0514	DNA polymerase III, epsilon subunit or related 3′-5′ exonuclease	+++	++	++	+++	++	No
Bbr_1224	*scpA*, segregation and condensation protein	++	++	+	++	+	No
Bbr_1370	Hypothetical protein, in operon with Uracil-DNA glycosylase	+++	++	+	+++	+	No
Bbr_1603	*radA*, DNA repair protein	+++	++	++	++	–	No
**REGULATORY PROTEINS**							
Bbr_0466	Hypothetical regulatory protein	+++	++	++	+++	++	No
Bbr_0589	Hypothetical regulatory protein, tetR family	+++	++	++	+++	+	No
**REQUIRE METAL ION AS COFACTOR**							
Bbr_0702	Haloacid dehalogenase-like hydrolase	+++	++	++	+++	++	No
Bbr_1743	Short chain dehydrogenase	+++	++	++	+++	++	No
**OTHER**							
Bbr_1644^d^	BD-type quinol oxidase subunit II; oxidoreductase	+++	++	++	+++	+	No
Bbr_1647	*glgX*, glycogen operon protein	+++	+++	+++	++	+	No

*Growth profiles of transposon mutants which were sensitive to iron chelation, selected for in RCM supplemented with 250 and 275 μM of 2′2-dipyridyl. A minus sign (−) indicates that final OD_600_ < 0.5; (+) indicates OD_600_ between 0.5 and 1.5, (++) indicates OD_600_ of 1.5 to 2.5 and (+++) indicates OD_600_ > 2.5*.

a *MTC for WT was found to be 275 μM of dipyridyl, two concentrations were used in the screening process in order to reduce the number of false positives obtained*.

b *MTC for WT was found to be 100 μM of ciclopirox olamine and 80 μM of phenanthroline*.

c *Genes which were differentially regulated in transcriptomic analysis of B. breve UCC2003 under iron limiting conditions*.

d *Two transposon insertional mutants were identified in this gene*.

e *Three transposon insertional mutants were identified in this gene*.

*B. breve* UCC2003-pBC1.2-bfeU-IR and *B. breve* UCC2003-pBC1.2-sifA-IR were prepared as follows for RNA extraction; Cells were incubated until an OD_600_ of 0.5 was achieved and were then exposed to 700 μM dipyridyl for 30 min, cells were harvested by centrifugation at 10,000 rpm for 2 min at room temperature and immediately frozen at −80°C. RNA extraction was carried out as previously described (Zomer et al., 2009).

The 5′ ends of the RNA transcripts were determined by annealing 1 pmol of an IRD700-labeled synthetic oligonucleotide to 20 μg of RNA, as previously described (Ventura et al., 2004). The following IRD700 labeled primer pairs were used: *bfeU*ifF and *bfeU*ifR, *sifA*ifF and *sifA*ifR (Table 3). Corresponding sequence ladders of the promoter regions of *bfeU* and *sifA* were produced using the same primer as in the primer extension reaction and employing a DNA cycle-sequencing kit (Jena Bioscience, Germany) and were run alongside the primer extension products to allow precise alignment of the transcriptional start site (TSS) with the corresponding DNA sequence. Separation was achieved on a 6.5% Li-Cor Matrix KB Plus acrylamide gel. Signal detection and image capture were performed employing a Li-Cor sequencing instrument (Li-Cor Biosciences).

### Murine colonisation experiments

Experiments with mice were approved by the University College Cork Animal Experimentation Ethics Committee and experimental procedures were conducted under license from the Irish Government (license number B100/3729). Seven-week-old female, BALB/c mice were housed in individually vented cages (Animal Care Systems) under a strict 12 h light cycle. Mice (*n* = 7 per group) were fed a standard polysaccharide-rich mouse chow diet and water *ad libitum*. Mice were inoculated by oral gavage 10^9^ cfu of *B. breve* UCC2003PK1, *B. breve* UCC2003-*bfeU* and *B. breve* UCC2003-*sifA* (see Table 1 for corresponding descriptions of strains). Faecal pellets were collected at intervals during 18 days to enumerate bacteria. Eighteen days after inoculation, mice were sacrificed and their intestinal tracts immediately dissected.

## Results

### Phenotypic screening for dipyridyl-sensitive mutants

A library containing ~20,000 *B. breve* UCC2003 random transposon mutants, previously constructed by Ruiz et al. (Ruiz et al., 2013), was screened in order to identify mutants with increased sensitivity to the ferrous iron chelator dipyridyl. The dipyridyl-sensitive phenotype was then validated for nearly 250 mutants by sub-cultivation in fRCM broth supplemented with dipyridyl (at a concentration of 250 or 275 μM), resulting in a final collection of 33 verified, dipyridyl-sensitive mutants. These 33 dipyridyl-sensitive mutants, were also analysed by growth profile analysis for their sensitivity two other iron chelators, ciclopirox olamine, a ferric iron chelator, and phenanthroline, an iron chelator which binds both ferrous and ferric iron (Bayer et al., 1964). The use of these three iron chelators allowed us to assess the cause of the growth defect of the obtained mutants as will be discussed below.

**Figure 1:**
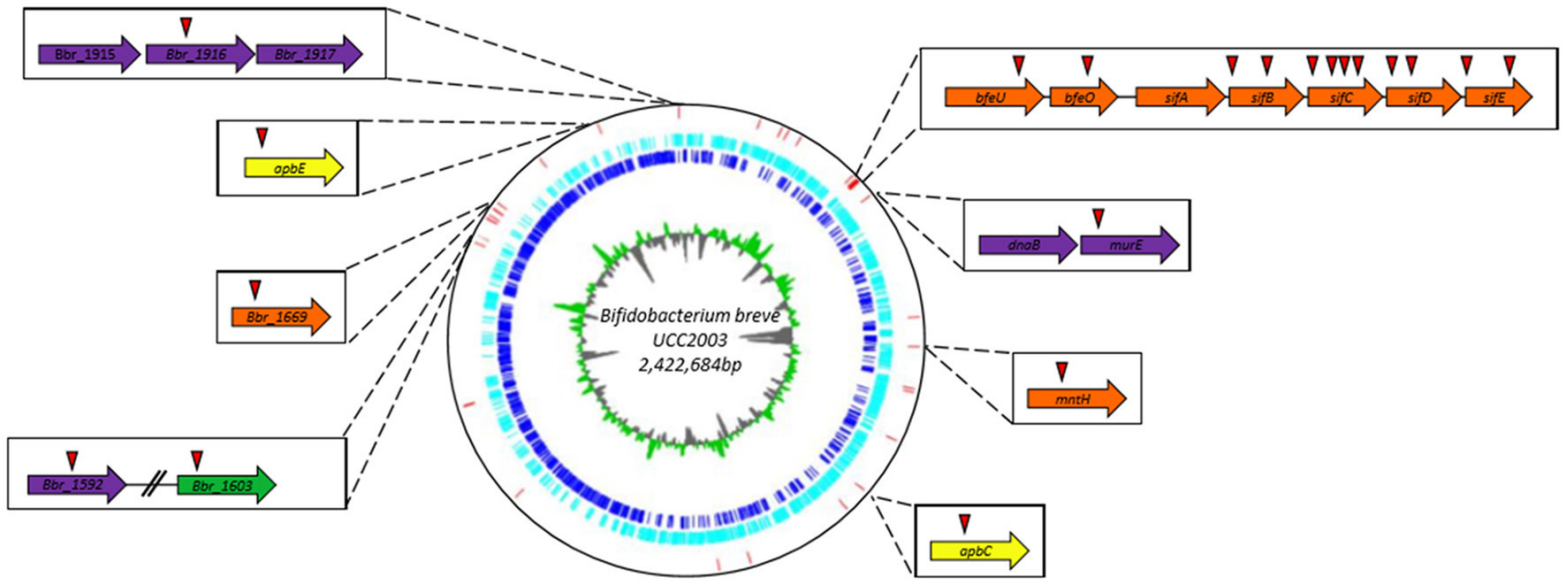
Circular genome map of *B. breve* UCC2003. The innermost circle illustrates GC skew, shown in green on the forward strand and in gray on the reverse strand. The dark blue circle displays the ORF on the forward strand, while the light blue circle representing all the ORF on the reverse strand. The red vertical bars indicate the transposon insertion sites of dipyridyl-sensitive mutants identified in the screening of the random mutant library. Genes in which transposon mutations were shown to cause complete growth impairment in the presence of either dipyridyl, phenanthroline or ciplopirox olamine are indicated in boxes together with their names or locus tags with the position(s) of the transposon insertion indicated by a red triangle.

The transposon insertion site of validated dipyridyl-sensitive mutants was then determined for these 33 mutants using an approach that was described previously (Ruiz et al., 2013). A list of these mutants can be found in Table 3 and Figure 1; some mutants had been isolated more than once, resulting in a final collection of 28 genes which were identified as important for survival under (ferrous) iron limiting conditions. Based on the predicted functions of the interrupted genes, the identified dipyridyl-sensitive mutants can be divided into seven functional groups (see Table 3): (i) transport associated, (ii) Fe-S cluster assembly associated, (iii) cell envelope associated, (iv) DNA repair/replication, (v) regulatory proteins, (vi) proteins which require a metal co-factor and (vii) other.

A number of genes encoding (based on BlastP and HHpred analysis) predicted transport systems were identified in the screening, namely Bbr_0427 (designated here as *mntH*), specifying a homolog of a divalent cation transporter in *E. coli*. The mutant identified in the *mntH* gene was found to be sensitive to dipyridyl and phenanthroline, yet was not sensitive to ferric iron chelation with ciclopirox olamine (at the concentration used for this chelator). A transposon insertion was also identified in Bbr_1816 (*oppC*), encoding a permease protein of an iron siderophore ABC transport system. Similar to the mutant in *mntH*, the strain carrying a mutation in *oppC* was shown to be sensitive to iron chelation with dipyridyl and phenanthroline, but not with ciclopirox olamine, indicating that both strains are specifically affected in ferrous iron uptake or metabolism.

Ten distinct mutants were identified, carrying Tn5 insertions in a gene cluster represented by locus tags Bbr_0221–Bbr_0227. The products of Bbr_0221-Bbr_0222 (*bfeUO*) are predicted to represent a high affinity ferrous iron uptake system which is similar to proteins encoded by (*efeUO*) of the *efeUOB* gene cluster found in *E. coli* (Grosse et al., 2006). Functional annotation analysis revealed that BfeU which contains nine transmembrane domains and encodes a conserved hypothetical membrane spanning protein with an iron permease FTR1 family domain. While BfeO encodes a conserved hypothetical protein, containing a hydrophobic N-terminus suggesting it is a membrane-associated protein that functions in metal ion binding. Interestingly, BfeO has homology to a human lactoferrin transporter of *Treponema pallidum* based on HHpred analysis. The deduced proteins of Bbr_0223-Bbr_0227, designated here as *sifABCDE*, are predicted to specify a ferrous iron/siderophore uptake system, based on similarities to previously characterised ferrous iron uptake/aerobactin siderophore uptake systems encoded by *E. coli* 1520, plasmid pIP1206 and *Bacillis subtilis* 168 (Cronin et al., 2012; Fukushima et al., 2013). Functional annotation analysis revealed that SifA is a conserved hypothetical membrane-spanning protein containing eight transmembrane domains and is homologous to a cation antiporter. SifB and SifC are ABC transporter permeases with FtsX-like family domains, while SifD is an ATP-binding subunit protein with homology to a ferric ion import ATP-binding subunit. SifE contains a hydrophobic N-terminus indicating that it is membrane associated, while it also contains an FMN-binding domain which may be associated with oxidoreductase activity. Interestingly, SifE is homologous to IsdH, a membrane bound protein which is involved in removing heme from hemoglobin in *Staphylococcus lugdunensis*. Mutations in the *bfeUO* gene cluster were shown to cause severely impaired growth in the presence of all three iron chelators, indicating that this predicted iron uptake system is important for the uptake of both ferrous and ferric iron. In contrast, mutations in the *sifABCDE* gene cluster were shown to negatively affect growth in medium supplemented with either dipyridyl or phenanthroline, whereas normal growth (compared to growth of UCC2003 under these conditions) was observed in the presence of ciclopirox olamine, indicating that this system is specifically responsible for the transport of ferrous iron (Table 3).

Furthermore, mutations identified in two genes involved in iron-sulfur (Fe-S) cluster formation, repair and docking were identified as causing severe growth impairment under iron limitation by dipyridyl. Fe-S clusters act as cofactors for many cellular proteins which have prominent roles in many cellular processes including but not limited to respiration, central metabolism, gene regulation, DNA repair and replication (Kiley and Beinert, 2003; Andreini et al., 2016). The first transposon mutant identified in a gene Bbr_1825 (designated here as *apbE*); *apbE* is homologous to *apbE* of *Salmonella enterica*, the product of this gene is known to play a role in the assembly and repair of Fe-S clusters (Skovran and Downs, 2003; Py and Barras, 2010). The second transposon insertion was identified was in a gene Bbr_0669 (designated as *apbC*), encoding a protein with similarity to a P-loop NTPase, which binds and transfers Fe-S clusters to cytosolic apo-proteins (Hausmann et al., 2005; Boyd et al., 2008). This *apbC*::*Tn5* mutant was severely impaired in growth in the presence of ferrous and ferric iron chelators, while the *apbE::Tn5* mutant was impaired in growth only in the presence of ferrous iron chelators.

A number of genes encoding cell envelope-associated proteins were identified in the phenotypic screen, namely: Bbr_0246, encoding a putative UDP-N-acetylmuramoylalanyl-D-glutamate–2,6-diaminopimelate ligase; Bbr_1592, encoding a putative lipid kinase; and Bbr_1916, which encodes a hypothetical secreted protein, and which is located in an operon with a gene specifying a predicted peptidoglycan lipid II flippase. These insertion mutants were impaired in growth in the presence of all iron chelators tested. Two genes with a predicted regulatory function were identified: Bbr_0589, a gene specifying a hypothetical TetR regulatory protein and Bbr_0466. The corresponding mutants were sensitive to ferrous iron but not ferric iron chelation. Two transposon insertions were identified in genes Bbr_1743 encoding a short chain dehydrogenase, and Bbr_0702 encoding a haloacid dehalogenase-like hydrolase; the products of these genes are reputed to require iron as a cofactor.

The remaining dipyridyl-sensitive mutants which were selected from the Tn5 library screening efforts, whose involvement in iron metabolism or requirement for iron is not immediately clear from similarity searches, are listed in Table 3.

### Transcriptomic response of *B. breve* UCC2003 to iron limitation

To gain additional insight into the genes important for *B. breve* UCC2003 survival under iron limiting conditions and to complement the results obtained through the phenotypic screening, microarray analysis was carried out. The transcriptomic response of *B. breve* UCC2003 upon iron limitation was assessed by subjecting cells in the exponential growth phase to a short but high concentration of dipyridyl (700 μM) for 30 min (Results are summarized in Table 4, complete microarray results can be found in Supplementary Tables S1, S2). Microarray analysis was carried out in this way in order to identify the immediate transcriptional response to acute and severe iron limitation. Genes with a significantly different transcription level relative to the control when grown under iron limiting conditions, were identified in this manner (*p* < 0.001 and an expression ratio of >1.7 or < 0.25). Based on these criteria a total of 57 genes were found to be differentially regulated under iron-limiting conditions.

**Table 4:** Effects of iron limitation on the transcriptome of *B. breve* UCC2003.

**Locus tag**	**Gene name and/or predicted Function**	**Upregulation**
Bbr_0579	Solute binding protein of ABC transporter system, iron siderophore, metallic cations	7.8
Bbr_0268	*silP*, Cation transport ATPase	6.7
Bbr_0222	*bfeO*, high affinity Fe^2+^ periplasmic transporter	3.7
Bbr_0826	SAM-dependent methyltransferase	3.3
Bbr_0221	*bfeU*, high-affinity Fe^2+^ permease	2.4
Bbr_1850	NADPH-dependent FMN reductase/Oxygen-insensitive NADPH nitroreductase	2.4
Bbr_0269	*csoR*, transcriptional regulator (copper-sensitive operon repressor)	2.1
Bbr_1817	*oppB*, permease protein of iron siderophore uptake system	1.9
Bbr_0223	*sifA*, hypothetical protein, possible siderophore binding protein	1.8
Bbr_0750	ATP-binding protein of ABC transporter system for metals	1.8
Bbr_0827	Hypothetical protein, containing cupin domain	1.8
Bbr_0225	*sifC*, permease protein ABC transporter ferric iron/siderophore uptake	1.7
Bbr_0224	*sifB*, permease protein ABC transporter ferric iron/siderophore uptake	1.7
Bbr_1815	*oppD*, ATP-binding protein of iron siderophore uptake system	1.7
**Locus tag**	**Gene name and/or predicted Function**	**Downregulation**
Bbr_1898	*nrdF*, Ribonucleoside-diphosphate reductase beta chain	4.8
Bbr_1899	*nrdE*, Ribonucleoside-diphosphate reductase alpha chain	4.2
Bbr_1104	*tsf*, Protein Translation Elongation Factor Ts (EF-Ts)	3.4
Bbr_1582	Narrowly conserved hypothetical membrane spanning protein	2.6
Bbr_0329	*atpD*, ATP synthase beta chain	2.5
Bbr_1446	*nrdG*, Anaerobic ribonucleoside-triphosphate reductase activating protein	2.4
Bbr_1622	*rplO*, 50S ribosomal protein L15	2.3
Bbr_1627	*rpsH*, 30S ribosomal protein S8	2.3
Bbr_1726	*rlpA*, LSU ribosomal protein L1P	2.3
Bbr_1623	*rpmD*, 50S ribosomal protein L30	2.2
Bbr_1628	*rpsN*, 30S ribosomal protein S14-1	2.2
Bbr_1626	*rplF*, 50S ribosomal protein L6	2.2
Bbr_1624	*rpsE*, 30S ribosomal protein S5	2.2
Bbr_1583	Histidine kinase sensor of two component system	2.1
Bbr_1632	*rpsQ*, 30S ribosomal protein	2.1
Bbr_1675	*rplL*, LSU ribosomal protein L12P (L7/L12)	2.1
Bbr_1581	Narrowly conserved hypothetical membrane spanning protein	2.1
Bbr_0899	Endonuclease involved in recombination	2.1
Bbr_0843	Conserved hypothetical secreted protein with excalibur domain	2.1
Bbr_0925	Permease MFS superfamily	2.0

One noteworthy feature of the *B. breve* UCC2003 transcriptome response to iron limitation was the transcriptional activation of most genes of the *bfeUO* and *sifABCDE* clusters, as discussed above. Furthermore, the gene cluster represented by locus tags Bbr_1817–Bbr_1814, predicted to encode an iron siderophore ABC transport system, was also upregulated under the imposed iron limiting conditions. Mutations in (some of the genes of) the *bfeUO/sifABCDE* and Bbr_1817–Bbr_1814 gene clusters cause a dipyridyl-sensitive phenotype (see above) and were the only genes to be identified using both of these approaches (i.e., phenotypic screening and microarray analysis).

Other genes encoding putative metal uptake systems were shown to be transcriptionally upregulated under the imposed iron-limiting conditions, including a solute binding protein of an ABC-type transport system (Bbr_0579). Based on BlastP and HHpred analysis this protein displays similarity to solute binding proteins for metal cations found in many species including *E. coli* and *Streptococcus pneumoniae* (Yatsunyk et al., 2008; Begg et al., 2015). The gene associated with locus tag Bbr_0268 (*silP*) displayed a 7.6-fold transcriptional increase under iron-limiting conditions. The protein product of *silP* contains an ATPase domain and is predicted to be involved in the transport of iron-carrying compounds and copper ions. Bbr_0269 (*csoR*) which was shown to be upregulated 2.1-fold under microarray analysis encodes a predicted copper-sensitive operon repressor.

Also of interest was the upregulation of Bbr_1850, a NADPH-dependent FMN reductase. Studies in *Pseudomonas putida* have found that these reductases have ferric iron reductase activity and are believed to play a role in NADH-dependent ferric/flavin reduction under iron stress conditions (Yeom et al., 2009). It is therefore possible that *B. breve* UCC2003 is utilising this reductase to convert cytosolic ferric iron or ferric iron bound to siderophore complexes into its more biologically available ferrous form for further use within the cell.

Additionally, a number of iron-dependent genes were downregulated in the microarrays, for example ribonucleoside-diphosphate reductase system (Bbr_1898 and Bbr_1899) and many other genes involved in purine and pyrimidine metabolism (Table 3). The ribonucleoside-diphosphate reductase system is essential for *de novo* synthesis of deoxy ribonucleotides, the precursors of DNA synthesis and consequently cell division.

### Transcriptional analysis of the *bfeUO/sifABCDE* gene cluster

**Figure 2:**
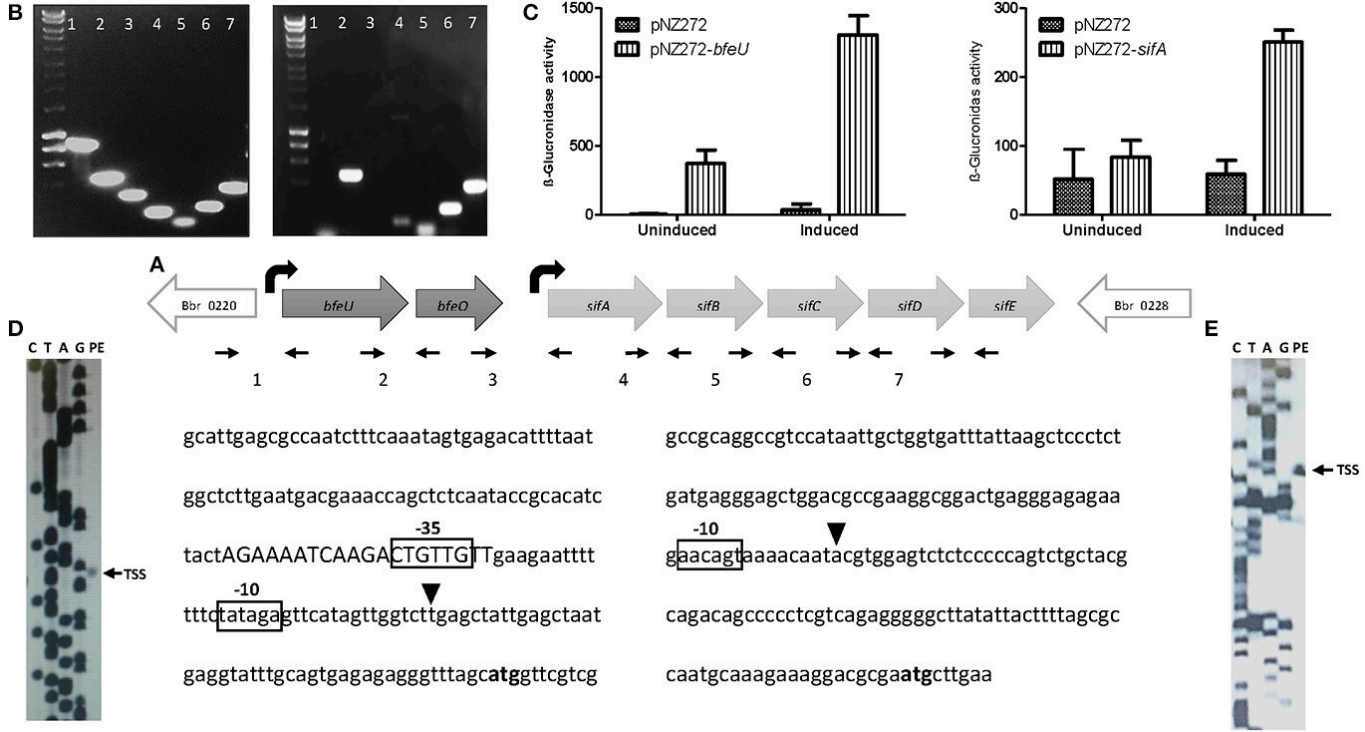
Transcriptional analysis of the *bfeUO/sifABCDE* gene cluster: **(A)** Schematic representation of the *bfeUO/sifABCDE* gene cluster, curved black arrows represent the predicted transcriptional start sites present, straight small arrows indicate positions of primers used. Intergenic region between genes *bfeU* to *sifE*, are labeled as follows; 1 (Bbr_0220/*bfeU*), 2 (*bfeU*/*bfeO*), 3 (*bfeO*/*sifA*), 4 (*sifA*/*sifB*), 5 (*sifB*/*sifC*), 6 (*sifC*/*sifD*), and 7 (*sifD*/*sifE*). **(B)** RT-PCR of the intergenic regions from Bbr_0220 to Bbr_0228, intergenic regions are labeled as in **(A)**. **(C)** β-glucuronidase assay of pNZ272+*bfeU*, pNZ272+*sifA* and pNZ272 (negative control). β-glucuronidase activity is expressed in Miller units. **(D)** Primer extension (PE) analysis of the transcriptional start site of *bfeU*. **(E)** PE analysis of transcriptional start site of *sifA*. In both **(D,E)** the start codon (ATG) is indicated in bold, the transcriptional start site (TSS) is indicated by a black triangle, the proposed −10 and −35 motifs are boxed and a possible operator sequence is displayed in uppercase.

Phenotypic screening of the random mutant library and microarray analysis showed that the *bfeUO/sifABCDE* (*bfe*/*sif*) cluster is transcriptionally induced and essential for normal growth of *B. breve* UCC2003 under iron-limiting conditions. A previous study on the *bfe* gene cluster of *B. breve* UCC2003 identified one promoter-containing region upstream of *bfeU* (Bbr_0221), which was used to create an inducible expression system for *Bifidobacterium* (Cronin et al., 2012). Due to the size of the intergenic region between the *bfeO*/*sifA* genes and the presence of a predicted promoter based on BPROM analysis we reasoned that *bfe/sif* region may encompass two transcriptional units. In order to assess this notion, RT-PCR was carried out across various sections of the *bfe/sif* gene cluster. Total RNA was extracted from logarithmically growing *B. breve* UCC2003 cultured in fRCM supplemented with dipyridyl (i.e., growth under iron-limiting conditions so as to ensure/maximize transcription of the *bfe/sif* gene cluster), and then used to generate cDNA by reverse transcription (see Materials and Methods). The resulting cDNA was used as a template for the amplification of the intergenic regions of *bfe/sif* gene cluster using various primer sets listed in Table 2. RT-PCR analysis indicated that the *bfe/sif* gene cluster indeed encompasses two transcriptional units represented by the *bfeUO* operon and the *sifABCDE* operon (Figure 2). Consistent with this analysis was the identification of a potential rho-independent transcriptional terminator sequence located downstream of *bfeO*, while the *sifABCDE* operon is followed by a gene, corresponding to locus tag Bbr_0228, which is oriented in the opposite direction (Figure 2).

The RT-PCR results indicated that transcription of the *bfeUO* and *sifABCDE* operons initiates upstream of the *bfeU* and *sifA* genes, respectively. A previous study had indicated that the *bfeU* promoter region was inducible under iron-limiting conditions, and in order to validate this and to investigate whether the promoter region upstream of *sifA* is also inducible under iron limiting conditions, plasmids pNZ-*bfeU* and pNZ-*sifA* were constructed, in which the upstream regions of *bfeU* and *sifA* were cloned in front of the promoter-less *gusA* gene in the promoter-probe vector pNZ272 (see Materials and Methods). *Plasmids* pNZ-*bfeU* and pNZ-*sifA*, as well as pNZ272 (negative control) were individually introduced into *B. breve* UCC2003, logarithmically growing cells of these three strains that either had or had not been subjected to iron limitation by means of dipyridyl addition were then utilised for β-glucuronidase (the product of *gusA*) activity assays. Consistent with our earlier study, we clearly demonstrate that each of the cloned fragments in pNZ-*bfeU* and pNZ-*sifA* elicit promoter activity and that these promoters are inducible by iron limitation (Figure 2, Cronin et al., 2012).

Next, given that the *bfeUO*/*sifABCDE* gene cluster encompasses two transcriptional units and that these units elicit promoter activity inducible by iron limitation, the transcriptional initiation sites were identified by primer extension analysis. This analysis identified a single TSS upstream of *bfeU* and *sifA*. The TSS of the *bfeUO* operon was shown to be located 48 bp upstream of the predicted *bfeU* start codon, and a putative promoter sequence was identified nine base pairs upstream of this TSS with putative −10 (CATAGT) and −35 (TTGAAG) elements which are similar to vegetative promoter sequences found in *B. breve* (Pokusaeva et al., 2010; O'Connell et al., 2014; Egan et al., 2015, 2016). The transcriptional start site of the *sifABCDE* operon was determined to be located 93 bp upstream of the *sifA* start codon, and a putative promoter sequence was identified eight base pairs upstream of this TSS with a putative −10 (AACAGT), though without any obvious −35 sequence (Figure 2). Our previous studies identified a putative regulatory motif (AAAATCAAGACTGTTGTT) upstream of *bfe*UO, which is also present upstream of a number of genes involved in iron utilization. The location of this binding operator sequence in relation to the *bfeUO* promoter region suggests that this as yet unidentified transcription factor may act as a repressor (Figure 2, Cronin et al., 2012).

### The presence of *bfeUO* or *sifABCDE* gene clusters is not required for colonisation of the healthy murine GIT

**Figure 3:**
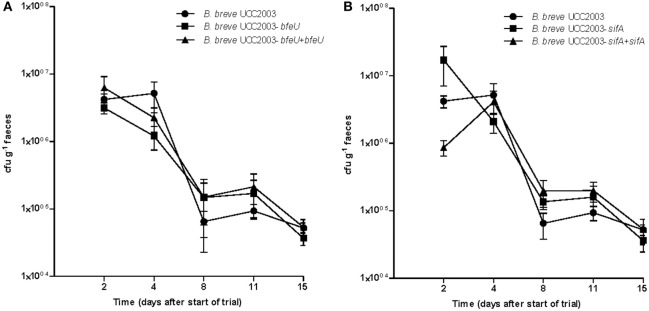
Murine colonisation trial: **(A)** illustrates the cfu g^−1^ faeces of *B. breve* UCC2003 (circle) and *B. breve* UCC2003-*bfeU* (square) and *B. breve* UCC2003-*bfeU*+pBC1.2+*bfeU* (triangle), while **(B)** illustrates cfu g^−1^ faeces of *B. breve* UCC2003 (circle), *B. breve* UCC2003-sifA (square), and *B. breve* UCC2003-sifA+pBC1.2+sifA (triangle) administered individually, administration started at day 0 and was continued for 3 consecutive days. Data shown are mean ± SEM. (*n* = 6).

Murine colonisation experiments were carried out to assess if *bfeUO*, a high affinity ferrous iron uptake cluster, or *sifABCDE*, a predicted ferrous iron/siderophore uptake system, play a role in gut colonisation. In order to analyse this the gut colonisation capacity of *B. breve* UCC2003, *B. breve* UCC2003-*bfeU, B. breve* UCC2003-*sifA* was tested in BALB/c mice. In conventional BALB/c mice with a resident microbiota (i.e., in a competitive environment), WT *B. breve* UCC2003, *bfeU* and the *sifA* insertion mutants were all able to colonise the gastrointestinal tract, as was shown by plating of fecal samples (1 × 10^5^ CFU/g feces retrieved 15 days after last administration; Figure 3). On the basis of these results it appears that the functionality of *bfeUO* and *sifABCDE* does not have any obvious impact on the ability of *B. breve* UCC2003 to colonise the gut of healthy BALB/c mice with a resident microbiota (Figure 3). These results are in agreement with previous work (Christiaen et al., 2014) who found that *B. breve* UCC2003-*bfeU* and *B. breve* UCC2003-*sifA* insertion mutant were able to colonise the gastrointestinal tract of nematodes as efficiently as the wild type strain.

### Distribution of the *bfeUO and sifABCDE* iron uptake systems across the *Bifidobacterium* genus

The importance of *bfeUO* and *sifABCDE* gene clusters under iron limitation has been clearly displayed through transcriptomic and phenotypic analysis, while transcriptional analysis clearly demonstrates that this gene cluster is composed of two transcriptional units *bfeUO* and *sifABCDE*. In order to gain more information about distribution and conservation of these gene clusters in the *Bifidobacterium* genus analysis utilising balstP was carried out. This was achieved by comparing the degree of protein similarity of the BfeUO and SifABCDE proteins found in *B. breve* UCC2003 with deduced protein complements of representative bifidobacterial species. The results are displayed in Table 5 as a heat map, which illustrates the protein similarity, as well as the genetic organisation and presence/absence of BfeUO- and SifABCDE-encoding genes across a number of bifidobacterial species. From this table it is obvious that the *bfeUO* and *sifABCDE* gene clusters are highly conserved across the bifidobacterial genus, and that these cluster are present in the genomes of 35 of the 47 bifidobacterial species tested. Interestingly the 12 bifidobacterial species which do not contain these gene clusters are members of three distinct branch points of a phylogenetic tree constructed by Milani et al. (2014). The members of these branch points are furthest away from the root of the evolutionary phylogenetic tree, which represents the *Bifidobacterium* genus ancestor (Milani et al., 2014). Furthermore, the five genes of the *sifABCDE* cluster are, when present, always co-located on the bifidobacterial genomes. The only exception to this was SifE, which encodes a predicted ferric reductase protein, and which appears to be absent from the genomes of *B. bohemicum* and *B. bombi*, bifidobacterial species that originate from the hind-gut of a bumblebee.

**Table 5:** Heat map representing the distribution and conservation of homologs of the BfeUO and SifABCDE iron uptake systems across the *Bifidobacterium* genus.

**Bifidobacterium genomes**	**Origin**	**Locus tag**	***bfeU***	***bfeO***	***sifA***	***sifB***	***sifC***	***sifD***	***sifE***
*Bifidobacterium actinocoloniiforme*	Bumblebee								
*Bifidobacterium adolescentis*	Adult faeces	BAD_0097-0103	63	67	69	66	70	90	67
*Bifidobacterium angulatum*	Adult faeces	BIANG_1097-1091	63	67	68	76	70	89	73
*Bifidobacterium animalis subsp. animalis*	Sewage								
*Bifidobacterium animalis subsp. lactis*	Fermented milk								
*Bifidobacterium asteroides*	Bee intestine	BAST_1624-1618	54	66	63	68	62	78	47
*Bifidobacterium biavatii*	Tamarind faeces	BBIA_1594-1587	82	81	84	87	91	93	67
*Bifidobacterium bifidum*	Infant faeces	BBPR_1727-1721	79	84	78	88	83	90	73
*Bifidobacterium bohemicum*	Bumblebee	BBOH_0687-0682	53	54	60	70	64	80	
*Bifidobacterium bombi*	Bumblebee	BBOMB_0050-0055	55	56	58	68	56	83	
*Bifidobacterium boum*	Bovine rumen	BBOU_1030-1024	59	78	69	72	71	85	53
*Bifidobacterium breve*	Infant faeces	Bbr_0221-0227	100	100	100	100	100	100	100
*Bifidobacterium callitrichos*	Marmoset faeces	BCAL_1913-1919	77	78	77	87	79	90	74
*Bifidobacterium catenulatum*	Adult faeces	BIFCAT_0140-0147	62	63	69	76	70	91	70
*Bifidobacterium choerinum*	Piglet faeces								
*Bifidobacterium coryneforme*	Bee intestine	BCOR_1391-1385	56	67	65	69	67	81	49
*Bifidobacterium crudilactis*	Raw milk cheese	BCRU_1683-1689	56	62	64	67	66	81	58
*Bifidobacterium cuniculi*	Rabbit faeces								
*Bifidobacterium dentium*	Oral cavity	BDP_0163-0169	65	67	70	76	71	91	67
*Bifidobacterium gallicum*	Human faeces								
*Bifidobacterium gallinarum*	Chicken caecum	BIGA_1581-1587	58	57	54	64	59	87	59
*Bifidobacterium indicum*	Bee intestine	BINDI_1328-1322	56	66	64	69	67	81	48
*Bifidobacterium kashiwanohense*	Infant faeces	BKAS_0947-0954	62	64	70	77	70	91	69
*Bifidobacterium longum subsp. infantis*	Infant faeces	Blon_0196-0202	83	93	94	95	93	99	89
*Bifidobacterium longum subsp. longum*	Adult faeces	BL_0455-0449	75	85	87	95	92	99	88
*Bifidobacterium longum subsp. suis*	Piglet faeces	BLSS_0743-0737	83	94	94	94	93	98	89
*Bifidobacterium magnum*	Rabbit faeces								
*Bifidobacterium merycicum*	Bovine rumen	BMERY_0538-0544	64	67	68	77	68	89	73
*Bifidobacterium minimum*	Sewage	BMIN_1327-1334	55	76	72	72	66	78	62
*Bifidobacterium mongoliense*	Fermented milk								
*Bifidobacterium pseudocatenulatum*	Infant faeces	BIFPSEUDO_4117-4125	62	63	69	76	70	91	68
*Bifidobacterium pseudolongum subsp. globosum*	Bovine rumen								
*Bifidobacterium pseudolongum subsp. pseudolongum*	Pig faeces								
*Bifidobacterium psychraerophilum*	Porcine caecum	BPSY_0711-0717	55	62	69	69	67	80	56
*Bifidobacterium pullorum*	Chicken faeces	BPULL_1164-1158	58	57	54	64	59	87	59
*Bifidobacterium reuteri*	Marmoset faeces	BREU_0405-0412	80	82	83	76	86	94	77
*Bifidobacterium ruminantium*	Bovine rumen	BRUM_0487-0493	63	68	71	70	70	90	66
*Bifidobacterium saeculare*	Rabbit faeces	BSAE_1410-1404	58	57	54	64	59	87	61
*Bifidobacterium sanguini*	Tamarind faeces	BISA_0498-0491	81	84	82	77	87	94	84
*Bifidobacterium scardovii*	Human sources	BSCA_0091-0085	88	91	91	89	89	93	77
*Bifidobacterium stellenboschense*	Tamarind faeces	BSTEL_1997-2003	79	81	78	86	80	94	79
*Bifidobacterium stercoris*	Adult faeces	BSTER_0114-0120	63	67	69	66	69	90	69
*Bifidobacterium subtile*	Sewage	BISU_0833-0838	58	46				79	47
*Bifidobacterium thermacidophilum subsp. porcinum*	Piglet faeces								
*Bifidobacterium thermacidophilum subsp. thermoacidophilum*	Anaerobic digester								
*Bifidobacterium thermophilum*	Piglet faeces	BTHER_1257-1263	59	78	69	71	71	85	52
*Bifidobacterium tsurumiense*	Hamster	BITS_0255-0249	57	74	65	72	66	88	54

*Heat map of the percentage protein identity of BfeUO and SifABCDE iron uptake systems from B. breve UCC2003 as compared with bifidobacterial representative species, Dark green shading represents > that 70% protein identity, green shading represents between 60 and 69% protein identity, light green shading below 60% identity and white shading indicates the absence of a gene*.

## Discussion

Bifidobacteria are believed to be able to propagate under low iron conditions and are thought to be efficient scavengers of iron, a notion being supported by the finding that certain bifidobacterial species are found in greater numbers under low luminal iron conditions as compared with normal or high luminal iron conditions. Much of the relevant literature to date focuses on the ability of bifidobacteria to internalise and sequester iron, and to proliferate in either high or low concentrations of iron (Bezkorovainy and Solberg, 1989; Kot et al., 1993; Kim et al., 2002; Vazquez-Gutierrez et al., 2015). The current study was aimed at uncovering the molecular mechanisms and systems responsible for iron uptake and metabolism in *B. breve* UCC2003, as a prototypical representative of its genus.

Severe iron limitation has a profound effect on *B. breve* UCC2003, as illustrated by the plethora of genes whose transcription is altered under such iron restrictive conditions and by the number of genes which were identified in the screening of the random mutant library under iron limiting conditions. This response includes the upregulation of Bbr_1850, a gene encoding a predicted NADPH-dependent FMN reductase. A similar reductase was identified to be involved in iron metabolism in *Pseudomonas putida*, and it has been speculated that such reductases may be needed in higher amounts to reduce ferric iron during iron limitation (Yeom et al., 2009; Takeda et al., 2010). Among the *B. breve* UCC2003 genes that were shown to be essential for growth under iron limiting conditions, we identified *apbC* and *apbE*, which are predicted to encode proteins required for Fe-S cluster formation, repair and docking. Mutations in homologs of *apbC* or *apbE* in *S. enterica* result in cellular deficiencies which are reversed by the addition of ferric chloride, which suggests that iron addition compensates for such mutations by increasing repair of oxygen-labile Fe-S clusters (Skovran and Downs, 2003). Therefore, in the case of *B. breve* UCC2003 it is possible that under iron limiting conditions mutation of *apbC* or *apbE* causes an inability for the cell to construct, repair or to load these Fe-S clusters into apo-proteins which are essential to many cellular process. This phenomenon, although not studied in full in this paper, would be of interest for future study.

Screening of the random mutant library and transcriptome analysis led to identification of several genes encoding factors for the transportation of cations and or siderophore complexes. From the current study it appears that there are two key uptake systems important for bifidobacterial survival under the imposed, *in vitro* iron-limiting conditions tested: BfeUO, a predicted high affinity ferrous/ferric iron uptake system and SifABCDE, a predicted Fe^2+^/siderophore uptake system, which specifically transports ferrous iron. BfeUO is similar to EfeUOB, a Fe^2+^ uptake system identified in *E. coli* and to a system present in *Bacillus subtilis*, which is responsible for ferrous and ferric iron transport depending on the extracellular conditions and the oxidant supply (Cao et al., 2007; Gaballa et al., 2008; Castells-Roca et al., 2011). This system was previously identified as BfeUO which was sensitive to both ferrous and ferric iron chelation, is highly conserved across the *Bifidobacterium* genus (Table 5) and is possibly the primary/predominant system responsible for the uptake of iron in *B. breve* UCC2003 under the *in vitro* iron limiting conditions tested here (Cronin et al., 2012).

SifABCDE represents a putative ferrous iron/siderophore uptake system, which was found to be important for *B. breve* UCC2003 survival when availability of ferrous iron is limiting. A relatively small number of siderophore uptake systems have been characterised in Gram-positive bacteria, however, of those characterised there are a number of common features, including a siderophore binding protein (possible SifA), ABC transporter permeases (SifB/C), ATPase (SifD), and a ferric iron reductase (SifE) (Fukushima et al., 2013, 2014). The identified mutants within the *bfeUO* gene cluster were sensitive to both ferrous and ferric iron chelation, while mutants located within the *sifABCDE* only exhibited sensitivity to ferrous iron chelation. This phenotype indicates that the *sifABCDE* cluster is specifically responsible for the uptake of free/complexed ferrous iron. Transcriptional analysis of *bfe*UO and *sifABCDE* via RT-PCR, and primer extension analysis found that the gene cluster is organised into two transcriptional units, which are subject to transcriptional induction upon iron limitation with dipyridyl. These results indicate that the expression of both the BfeUO and SifABCDE uptake systems is induced under ferrous iron limitation, while phenotypic analysis indicates that that the sifABCDE uptake system is specifically responsible for the uptake of ferrous iron, the form of iron that is expected to more prevalent in the anaerobic GIT.

A recent study carried out by Vazquez-Gutierrez et al. identified a number of iron uptake clusters utilising a proteomic approach, this analysis identified three ferrous iron transporters in the secretome of *B. kashiwanohense* PV20-2: AH68_00590 and AH68_00595 which are homologous to *bfeUO*, respectively, AH68_00600 which is homologous to *sifA* (Vazquez-Gutierrez et al., 2017). The presence of BfeUO and SifA homologs in these three bifidobacterial strains illustrate the importance of these two iron uptake system in sequestering this vital micronutrient. From the phylogenetic analysis illustrated in Table 5 it is also evident that these iron uptake systems are widely distributed and are highly conserved across the *Bifidobacterium* genus. Furthermore, the identification of a number of other potential cation uptake systems such as MntH and that encoded by Bbr_1817–1814 illustrates the diversity and possible complementarity of iron sequestration mechanisms within the *Bifidobacterium* genus.

Previously *Bifidobacterium* species have been found to operate a LuxS-mediated system for gut colonisation and pathogen protection, which is linked to iron acquisition (Christiaen et al., 2014). Similar to the findings of the murine colonisation experiments carried out in this study, Christiaen et al. found that *bfeU* and *sifA* insertion mutants are able to colonise the GIT of nematodes as efficiently as the WT strain. In addition, these authors found that the *bfeU* and *sifA* mutants exhibit a significantly decreased ability to confer protection to *Salmonella*-infected nematodes as compared to the WT strain (Christiaen et al., 2014). Deriu et al. have elegantly demonstrated that the fecal iron concentration of *S. thyphimurium* infected mice is reduced to 300 mg/kg from 950 mg/kg in the absence of infection. Therefore, confirming that infection causes a reduction of iron in the gut. They also illustrate how the probiotic strain *E. coli* Nissle reduces growth and intestinal colonisation of *S. typhimurium* by competing for iron in the colonic environment (Deriu et al., 2013). Consistent with this observation, it was also demonstrated that two bifidobacterial strains; *B. pseudolongum* PV8-2 and *B. kashiwanohense* PV20-2 exhibit strain-dependent inhibitory activity on iron-dependent enteropathogens (Vazquez-Gutierrez et al., 2016). The fact that insertion mutations within these uptake systems (*bfeUO* and *sifABCDE*) do not affect the colonisation efficiency of *B. breve* UCC2003 in the healthy nematode or murine gut leads us to believe that iron availability in a healthy gut is sufficient for colonisation of *B. breve* UCC2003, *B. breve* UCC2003-*bfeU* or *B. breve* UCC2003-*sifA*, even though the latter two mutants exhibit an *in vitro* growth deficiency under iron-limiting conditions. Therefore, we postulate that either (sufficient) iron is available in the gut in a form which is not taken up by the *bfeUO* or *sifABCDE*, or that these systems for iron acquisition may be important for *B. breve* UCC2003 survival during times in which iron is more limiting, for example during GIT infection as demonstrated by Deriu et al. (2013).

Altogether this study has also helped to broaden our knowledge regarding *Bifidobacterium* response to iron limitation, with the identification of a variety of genes being important for *B. breve* UCC2003 survival under iron-limiting conditions. This study also demonstrates the effectiveness of utilising random mutagenesis as a tool for the exploration of genes involved in iron metabolism and uptake in *Bifidobacterium*. The characterisation of the *sifABCDE* and *bfeUO* iron uptake cluster revealed that these clusters are vitally important *B. breve* UCC2003 survival under ferric and ferrous iron chelation, of which the latter is the most predominant in the gastrointestinal environment. Importantly, the *sifABCDE* iron uptake cluster was identified as being specifically responsible for the uptake of ferrous iron, while the *bfeUO* iron uptake system was sensitive to both ferrous and ferric iron chelation. Taken together, this versatile response of bifidobacteria to iron limitation may be one of the factors that afford such gut commensals a competitive edge among the many microbes within the gastrointestinal tract.

## Ethics statement

Animal procedures performed in this study were approved by the UCC Animal Experimentation Ethics Committee and were regulated the Department of Health of the Irish Government. All animal housing and procedures conformed to relevant Irish and European legislation governing the use of laboratory animals for scientific experimentation.

## Author contributions

NL, Conceived and designed experiments, Carried out experiments, Analysed data, Wrote paper, Technical and scientific discussion. FB, Carried out experiments, Analyzed data, Technical and scientific discussion. PC, Carried out experiments. MO, Carried out experiments. DV, Conceived and designed experiments, Analyzed data, Wrote paper, Technical and scientific discussion.

### Conflict of interest statement

The authors declare that the research was conducted in the absence of any commercial or financial relationships that could be construed as a potential conflict of interest.
