# Supplementary material for: Genome-Wide Search for Genes Required for Bifidobacterial Growth under Iron-Limitation
Source: Front Microbiol. 2017 May 31;8:964. doi: 10.3389/fmicb.2017.00964 (PMC5449479; doi:10.3389/fmicb.2017.00964)
Supplement: Supplementary file 1 [file Table1.DOCX]

| **Locus tag** | **Upregulation** | **Gene name and/or predicted Function** |
| --- | --- | --- |
| Bbr_0579 | 7.6 | Solute binding protein of ABC transporter system, iron siderophore, metallic cations |
| Bbr_0268 | 7.2 | *silP*, Cation transport ATPase |
| Bbr_0222 | 3.9 | *bfeO*, Conserved hypothetical secreted protein |
| Bbr_0826 | 3.2 | SAM-dependent methyltransferase |
| Bbr_0433 | 3.0 | Transposase |
| Bbr_1774 | 3.0 | Transposase |
| Bbr_0221 | 2.5 | *bfeU*, High-affinity Fe^2+^ permease |
| Bbr_1850 | 2.5 | NADPH-dependent FMN reductase/Oxygen-insensitive NADPH nitroreductase |
| Bbr_0312 | 2.4 | Transposase |
| Bbr_1401 | 2.1 | Transposase |
| Bbr_0302 | 2.1 | Transposase |
| Bbr_1710 | 2.1 | *rbsK5*, Ribokinase |
| Bbr_1328 | 2.0 | Bbr_1328 Conserved hypothetical membrane spanning protein |
| Bbr_0269 | 2.0 | *csoR* , transcriptional regulator (copper-sensitive operon repressor) |
| Bbr_1822 | 1.9 | Narrowly conserved hypothetical protein |
| Bbr_0348 | 1.9 | *ansB, a*spartate ammonia-lyase |
| Bbr_1870 | 1.9 | *mesJ,* tRNA-specific adenosine deaminase |
| Bbr_0107 | 1.9 | *cebF*, Cellobiose/cellotriose transport system permease protein CebF |
| Bbr_0108 | 1.9 | *cebG,* Cellobiose/cellotriose transport system permease protein CebG |
| Bbr_1817 | 1.9 | *oppB*, Cation/iron containing molecules transporter system permease protein |
| Bbr_0099 | 1.8 | *scrR1,* Transcriptional regulator, LacI family |
| Bbr_0106 | 1.8 | *cebE,* Cellobiose/cellotriose binding protein |
| Bbr_0225 | 1.8 | *sifC*, permease protein ABC transporter ferric iron/siderophore uptake |
| Bbr_1720 | 1.8 | *accD,* Acetyl-/propionyl-CoA carboxylase beta chain |
| Bbr_1545 | 1.8 | Mobilisation protein |
| Bbr_0024 | 1.8 | Transposase |
| Bbr_0827 | 1.8 | Conserved hypothetical protein containing cupin domain |
| Bbr_0110 | 1.8 | *ilvC2* Ketol-acid reductoisomerase/2-dehydropantoate 2-reductase |
| Bbr_0750 | 1.8 | ATP-binding protein of ABC transporter system for metals |
| Bbr_0109 | 1.7 | *bgl1,* Beta-glucosidase |
| Bbr_0223 | 1.7 | *sifA*, hypothetical protein, possible siderophore binding protein |
| Bbr_0758 | 1.7 | *aspA,* ADP-ribose pyrophosphatase |
| Bbr_0087 | 1.7 | Conserved hypothetical membrane spanning protein (hemolysin III homolog) |
| Bbr_0224 | 1.7 | *sifB*, Permease protein ABC transporter ferric iron/siderophore uptake |
| Bbr_1527 | 1.7 | Narrowly conserved hypothetical membrane spanning protein |
| Bbr_1827 | 1.7 | ATP-binding and permease protein of ABC transporter system |
| Bbr_1403 | 1.7 | Transposase |
| Bbr_1539 | 1.7 | Conserved hypothetical protein |
| Bbr_1543 | 1.7 | Hypothetical protein |
| Bbr_1815 | 1.7 | *oppD,* cation/iron containing molecules transport ATP-binding protein |
| Bbr_0129 | 1.7 | *fabG* 3-oxoacyl-[acyl-carrier protein] reductase |
| Bbr_0089 | 1.7 | *degP,* DO serine protease containing PDZ domain |
| Bbr_0252 | 1.7 | Conserved hypothetical protein with alanine racemase, N-terminal domain |
| Bbr_1814 | 1.7 | *oppA,* cation/iron containing molecules binding protein |

**Supplementary Table S1**: B. breve **UCC2003 genes up regulated in expression during growth in fRCM supplemented with 700µM dipyridyl**

The level of expression is shown as a fold-value of increase in expression, with a cut-off of a minimum >1.7-fold increase in expression.
