# Supplementary material for: Genome-Wide Search for Genes Required for Bifidobacterial Growth under Iron-Limitation
Source: Front Microbiol. 2017 May 31;8:964. doi: 10.3389/fmicb.2017.00964 (PMC5449479; doi:10.3389/fmicb.2017.00964)
Supplement: Supplementary file 2 [file Table2.DOCX]

Table S2: B. breve **UCC2003 genes down regulated in expression during growth in fRCM supplemented with 700µM dipyridyl**

| **Locus tag** | **Down Regulation** | | **Gene name and/or predicted Function** |
| --- | --- | --- | --- |
| Bbr_1898 | 4.5 | *nrdF,* Ribonucleoside-diphosphate reductase beta chain | |
| Bbr_1899 | 3.9 | *nrdE,* Ribonucleoside-diphosphate reductase alpha chain | |
| Bbr_1104 | 3.4 | *tsf,* Protein Translation Elongation Factor Ts (EF-Ts) | |
| Bbr_1582 | 2.6 | Narrowly conserved hypothetical membrane spanning protein with PspC domain | |
| Bbr_1446 | 2.4 | *nrdG,* Anaerobic ribonucleoside-triphosphate reductase activating protein | |
| Bbr_0329 | 2.3 | *atpD,* ATP synthase beta chain | |
| Bbr_1622 | 2.3 | *rplO,* 50S ribosomal protein L15 | |
| Bbr_1726 | 2.2 | *rlpA,* LSU ribosomal protein L1P | |
| Bbr_1583 | 2.2 | Histidine kinase sensor of two component system | |
| Bbr_1623 | 2.1 | *rpmD,* 50S ribosomal protein L30 | |
| Bbr_1627 | 2.1 | *rpsH,* 30S ribosomal protein S8 | |
| Bbr_0899 | 2.1 | Endonuclease involved in recombination | |
| Bbr_1581 | 2.1 | Narrowly conserved hypothetical membrane spanning protein | |
| Bbr_0843 | 2.1 | Conserved hypothetical secreted protein with excalibur domain | |
| Bbr_0925 | 2.1 | Permease MFS superfamily | |
| Bbr_1632 | 2.0 | *rpsQ,* 30S ribosomal protein S17 | |
| Bbr_1628 | 2.0 | *rpsN,* 30S ribosomal protein S14-1 | |
| Bbr_1626 | 2.0 | *rplF,* 50S ribosomal protein L6 | |
| Bbr_1228 | 2.0 | *rplT,* LSU ribosomal protein L20P | |
| Bbr_1633 | 2.0 | *rpmC,* 50S ribosomal protein L29 | |
| Bbr_1675 | 2.0 | *rplL,* LSU ribosomal protein L12P (L7/L12) | |
| Bbr_1624 | 2.0 | *rpsE,* 30S ribosomal protein S5 | |

The level of expression is shown as a fold-value of increase in expression, with a cut-off of a minimum >2-fold increase in expression.
